# Targeting CD38 with monoclonal antibodies disrupts key survival pathways in paediatric Burkitt's lymphoma malignant B cells

**DOI:** 10.1002/cti2.70011

**Published:** 2024-10-03

**Authors:** Kathrin Kläsener, Nadja Herrmann, Liliana Håversen, Timothy Sundell, Martina Sundqvist, Christina Lundqvist, Paul T Manna, Charlotte A Jonsson, Marcella Visentini, Diana Ljung Sass, Sarah McGrath, Kristoffer Grimstad, Alaitz Aranburu, Karin Mellgren, Linda Fogelstrand, Huamei Forsman, Olov Ekwall, Jan Borén, Inger Gjertsson, Michael Reth, Inga‐Lill Mårtensson, Alessandro Camponeschi

**Affiliations:** ^1^ Department of Rheumatology and Clinical Immunology University Medical Center Freiburg Freiburg Germany; ^2^ Signaling Research Centers BIOSS and CIBSS University of Freiburg Freiburg Germany; ^3^ Department of Rheumatology and Inflammation Research, Institute of Medicine, Sahlgrenska Academy University of Gothenburg Gothenburg Sweden; ^4^ Department of Molecular and Clinical Medicine, Institute of Medicine, Sahlgrenska Academy University of Gothenburg Gothenburg Sweden; ^5^ Department of Physiology, Institute of Neuroscience and Physiology, Sahlgrenska Academy University of Gothenburg Gothenburg Sweden; ^6^ Department of Translational and Precision Medicine Sapienza University of Rome Rome Italy; ^7^ Department of Pediatric Hematology and Oncology, The Queen Silvia's Hospital for Children and Adolescents University of Gothenburg Gothenburg Sweden; ^8^ School of Bioscience University of Skövde Skövde Sweden; ^9^ Department of Laboratory Medicine, Institute of Biomedicine, Sahlgrenska Academy University of Gothenburg Gothenburg Sweden; ^10^ Department of Clinical Chemistry Sahlgrenska University Hospital Gothenburg Sweden; ^11^ Department of Pediatrics, Institute of Clinical Sciences, Sahlgrenska Academy University of Gothenburg Gothenburg Sweden; ^12^ Department of Rheumatology, Region Västra Götaland Sahlgrenska University Hospital Gothenburg Sweden; ^13^ Department of Clinical Immunology and Transfusion Medicine, Region Västra Götaland Sahlgrenska University Hospital Gothenburg Sweden

**Keywords:** CD38‐targeting monoclonal antibodies, chemotherapy resistance, daratumumab, immunotherapy, isatuximab, paediatric Burkitt's lymphoma

## Abstract

**Objectives:**

Paediatric Burkitt's lymphoma (pBL) is the most common childhood non‐Hodgkin B‐cell lymphoma. Despite the encouraging survival rates for most children, treating cases with relapse/resistance to current therapies remains challenging. CD38 is a transmembrane protein highly expressed in pBL. This study investigates the effectiveness of CD38‐targeting monoclonal antibodies (mAbs), daratumumab and isatuximab, in impairing crucial cellular processes and survival pathways in pBL malignant cells.

**Methods:**

*In silico* analyses of patient samples, combined with *in vitro* experiments using the Ramos cell line, were conducted to assess the impact of daratumumab and isatuximab on cellular proliferation, apoptosis and the phosphoinositide 3‐kinase (PI3K) pathway.

**Results:**

Isatuximab was found to be more effective than daratumumab in disrupting B‐cell receptor signalling, reducing cellular proliferation and inducing apoptosis. Additionally, isatuximab caused a significant impairment of the PI3K pathway and induced metabolic reprogramming in pBL cells. The study also revealed a correlation between CD38 and MYC expression levels in pBL patient samples, suggesting CD38 involvement in key oncogenic processes.

**Conclusion:**

The study emphasises the therapeutic potential of CD38‐targeting mAbs, particularly isatuximab, in pBL.

## Graphical abstract

This study identifies CD38 as a potential target for treating paediatric Burkitt's lymphoma. We compare two monoclonal antibodies targeting CD38, finding isatuximab more effective than daratumumab in reducing cell proliferation, inducing apoptosis and impacting key survival pathways in lymphoma cells.

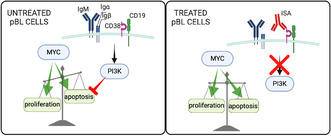

## Introduction

Paediatric Burkitt's lymphoma (pBL) is an aggressive cancer and the prevalent form of non‐Hodgkin's B‐cell lymphoma in childhood.^1^ It is characterised by rapidly growing tumors arising from B cells and can occur in different parts of the body. Treatment typically involves chemotherapy and immunotherapy, including monoclonal antibodies (mAbs) such as rituximab targeting CD20. Despite therapeutic advancements, available treatments do not cure approximately 10–20% of patients, highlighting the urgency for alternative therapies.^2^ A defining characteristic of pBL is the chromosomal translocation [*t*(8;14)] that places the oncogene *MYC* downstream of the active promoter of the *IGH* chain locus or, less commonly, the *IGL* chain loci, either *IGK* [*t*(2;8)] or *IGL* [*t*(8;22)], resulting in high *MYC* expression.^3^, ^4^ Despite these translocations, pBL cells express on their surface a B‐cell antigen receptor (BCR), often IgM.

CD38 is a transmembrane protein highly expressed on the surface of numerous malignancies, including pBL.^5^ In B‐cell chronic lymphocytic leukaemia (B‐CLL), CD38 expression predicts poor prognosis.^6^ In multiple myeloma, CD38‐targeting mAbs, such as daratumumab (DARA), have been approved for treatment.^7^ In the context of pBL, the potential of CD38 as a target for therapeutic intervention remains unclear.

Previous studies have shown that CD38 ligation in B‐cell progenitors, which lack the expression of a BCR on the cell surface, triggers tyrosine phosphorylation of the cytoplasmic tail of CD19, a co‐receptor of the BCR in mature B cells.^8^ In a study on B‐CLL, CD38 was observed to colocalise with components of the BCR complex, including IgM, Igα and Igβ, as well as with CD19 and CD81, another co‐receptor of the BCR.^9^ Moreover, when engaged by mAbs, cross‐linkers or its ligand CD31, CD38 relocalises to cap areas alongside the CD19/CD81 complex, highlighting its dynamic association with these molecules.^10^ We have recently shown that indeed CD38 forms a complex on the surface of B cells with CD19.^11^ When a BCR recognises an antigen, it triggers the phosphorylation of tyrosine‐based activation motifs in the cytoplasmic domain of the Igα/Igβ signal transduction heterodimer linked to the BCR, initiating a signalling cascade that leads to B‐cell activation.^12^ In resting B cells, the BCR transmits low levels of ‘tonic’ signals that support cell survival, partly mediated by CD19.^13^ The cytoplasmic tail of CD19 contains tyrosine residues that provide docking sites for phosphoinositide 3‐kinase (PI3K), which plays a central role in tonic signalling survival in B cells. The PI3K pathway also supports proliferation and survival of pBL cells and has been shown to confer chemotherapy resistance in several tumor types.^14^, ^15^ Furthermore, constitutive activation of this pathway is associated with the development of treatment resistance in pBL cell lines.^16^ Indeed, in pBL B cells, BCR and CD19 signalling through PI3K is constant and independent of antigens, resembling ‘tonic’ signalling.^17^ The MYC protein not only supports cell proliferation but also induces apoptosis. In pBL, the pro‐survival signal provided by the PI3K pathway counterbalances this apoptotic effect.^14^

CD19 and IgM are organised as nanoclusters in distinct cell surface compartments.^18^ Upon B‐cell activation, CD19 and IgM come into close proximity, thereby enhancing PI3K signalling.^19^ We found that IgM:CD19 interaction is disrupted by treatment with DARA, impairing proliferation and survival of pBL cell lines.^11^ Our previous findings, combined with the central role that PI3K plays in pBL cells, motivated our exploration into whether targeting CD38 could reduce the activity of PI3K in pBL. In this study, we used the Ramos cell line, which is derived from a patient with pBL and harbours the *IGH/MYC* translocation, as our model for pBL. We evaluated the effects on Ramos cells of DARA and isatuximab (ISA), the latter being a recently approved mAb for treating relapsed multiple myeloma.^20^ We assessed proliferation, apoptosis, cell cycle, intracellular calcium (Ca^2+^) flux, metabolism, interactions of surface proteins such as IgM and CD19, and activation of downstream pathways by quantifying the phosphorylation of key kinases, that is spleen tyrosine kinase (SYK) and AKT, also known as protein kinase B. Together with our crystal structure analyses of CD38's ectodomain and its resulting conformational changes upon mAbs binding, our study suggests that targeting CD38 holds therapeutic potential for treating malignant pBL cells and that ISA is more effective than DARA in this context.

## Results

### *CD38* correlation with *MYC* in pBL samples

**Figure 1:**
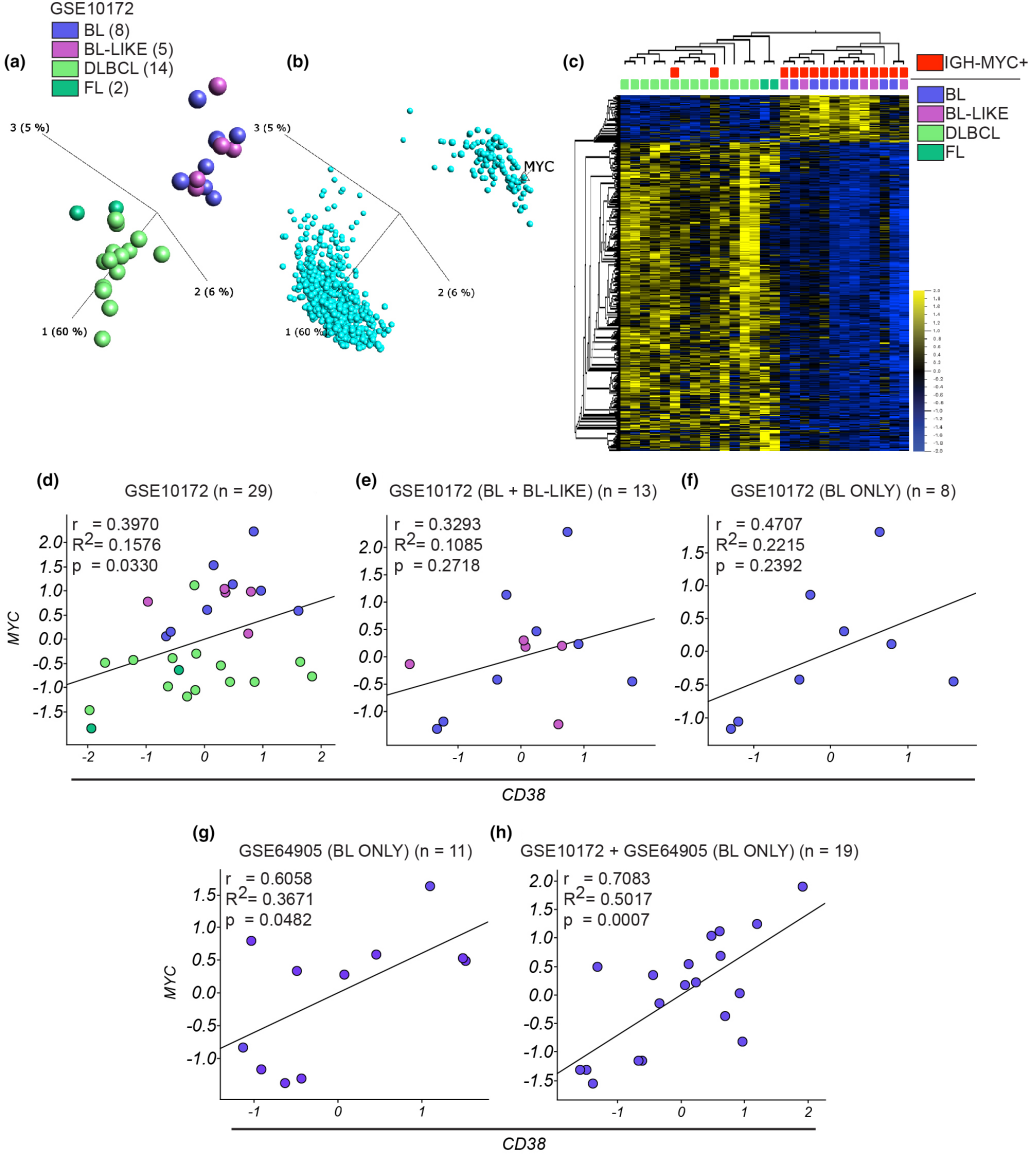
Correlation analysis of *CD38* and *MYC* gene expression in pBL patient samples. **(a)** Unsupervised PCA analysis shows the cluster pattern of the 29 paediatric lymphoma samples (GSE10172) based on significantly differentially expressed genes among these samples (1800 genes, one‐way ANOVA with the post hoc Tukey test *P* = 0.01); the colour key for disease subtypes in this panel is consistent with those used in the following panels of this figure. **(b)** PCA chart shows the distribution of the significantly differentially expressed genes that lead to the samples clustering in **a**. **(c)** Heatmap shows the 1800 significantly differentially expressed genes and the hierarchical distribution of the samples reflecting the clustering in **a**. The disease subtype and absence/presence of the IgH‐MYC translocation related to these samples are also reported. Correlation analysis for *MYC* and *CD38* gene expression was performed on the samples contained in the dataset GSE10172 and Pearson's correlation coefficient (*r*), coefficient of determination (*R* ^2^) and *P*‐value were calculated for **(d)** the whole 29 samples contained in the data set, **(e)** only for BL and BL‐like samples, **(h)** only for BL samples. **(g)** Same analysis as in **(d–f)** but on 11 pBL samples contained in the GSE64905 dataset. **(h)** Same analysis as in **(d–g)** but on 19 pBL samples contained in GSE10172 and GSE64905 data sets. Here, the data sets were merged and normalised using the *Z*‐score normalisation method.

A recent study assessed CD38 protein expression on the cell surface of paediatric haematological malignancies to establish the basis for CD38‐targeting mAb therapies and pBL showed the highest levels.^5^ Furthermore, findings from another study indicated that high CD38 protein expression is a significant indicator of *MYC* rearrangement in high‐grade B‐cell lymphomas, a category that includes aggressive lymphomas such as BL.^21^ This study also reported that patients with *MYC* rearrangements tended to be younger than those without, reflecting the higher prevalence of BL, a *MYC*‐driven disease, in paediatric malignacies. In the light of these findings, we analysed online gene expression data sets of pBL samples and compared them with other paediatric lymphomas to investigate the *CD38* gene expression profile and its correlation with *MYC* gene expression. The data set GSE10172 includes samples from eight children diagnosed with BL, five BL‐like, 14 diffuse large B‐cell lymphoma (DLBCL) and two follicular lymphoma (FL).^22^ Unsupervised principal component analysis clustered BL with BL‐like and DLBCL with FL (Figure 1a). BL‐like has characteristics similar to BL and may harbour the *IGH‐MYC* translocation.^23^ Indeed, the genes contributing to the clustering shown in Figure 1a revealed *MYC* as a pivotal gene (Figure 1b). A hierarchical clustering analysis including genetic information of these samples showed that BL and BL‐like presented a similar gene expression profile and all BL and BL‐like samples harboured the *IGH‐MYC* translocation (Figure 1c). This was also confirmed by the high level of *MYC* expression in these samples compared with DLBCL and FL (Supplementary figure 1a). *CD38* gene expression showed a more pronounced and uniform pattern in BL and BL‐like, although with no significant differences, whereas DLBLC displayed a broader range of expression levels (Supplementary figure 1b). As we hypothesised that targeting CD38 with mAbs may alter MYC functions, we investigated whether there was a correlation between *CD38* and *MYC* expression levels in pBL samples. Analysis of all the samples included in GSE10172 revealed a significant but moderate positive correlation with a coefficient (*r*) of 0.397 (Figure 1d). The coefficient of determination (*R* ^2^) was 0.1576, that is approximately 16% of the variance in one variable could be explained by the other variable. When we restricted our analysis to BL and BL‐like samples within GSE10172, this positive correlation diminished and became statistically non‐significant (Figure 1e). Further narrowing our focus to the BL samples revealed a slightly stronger positive correlation (*r* = 0.47, *R* ^2^ = 0.22), but it remained not statistically relevant, likely because of the limited sample size (Figure 1f). Turning our attention to a different data set, GSE64905, composed of a larger collection of only pBL samples (*n* = 11), we found a significant positive correlation (*r* = 0.60, *R* ^2^ = 0.3671) (Figure 1g).^24^ Integration of the pBL samples data from the two data sets resulted to an even more significant and robust positive correlation (*r* = 0.70, *R* ^2^ = 0.50) (Figure 1h). These results support a potential functional involvement for CD38 in the oncogenic mechanisms driven by MYC in pBL, making CD38 a valid target for immunotherapies in this context.

### Conformational alteration of CD38 ectodomain by ISA binding and cyclase activity inhibition

**Figure 2:**
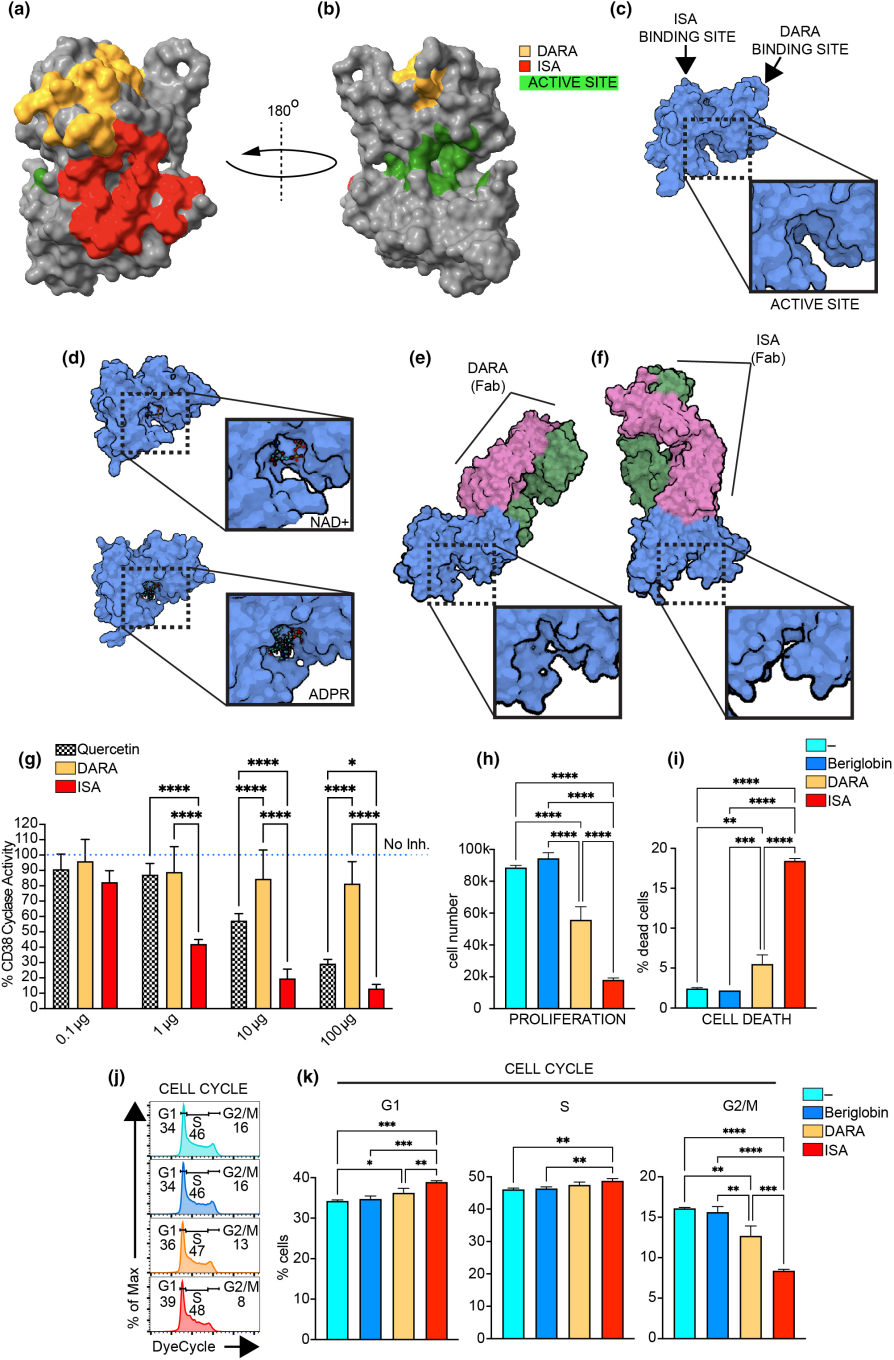
DARA and ISA differential interaction with CD38's structure and its cyclase activity and impact on pBL cell proliferation, apoptosis and cell cycle. **(a, b)** The extracellular domain's structure of CD38 from two perspectives (top and 180° rotated bottom view, respectively), highlighting the epitopes recognised by DARA and ISA and the active site's location. **(c)** The crystal structure of the unbound CD38 ectodomain (PDB entry: 1YH3), showing the accessible active site. Arrows indicate the predicted binding sites for DARA and ISA. The dashed box indicates active site. A zoomed‐in view of the active site is provided in the adjacent box. **(d)** The crystal structure of CD38's ectodomain complexed with NAD^+^ (upper panel) and ADPR (lower panel) in its active site (PDB entries: 3OFS and 8P8C respectively). The dashed box indicates active site, NAD^+^ and ADPR. A zoomed‐in view of the active site is provided in the adjacent box. **(e, f)** The conformational changes of CD38 when bound to the Fab region of DARA (PDB entry: 7DHA) and ISA (PDB entry: 4CMH), respectively. Dashed boxes indicate the active site. A zoomed‐in view of the active site is provided in the adjacent boxes. **(g)** Concentration‐dependent inhibition of CD38 cyclase activity by DARA, ISA and quercetin, a CD38 cyclase inhibitor. The assay was performed on recombinant CD38 protein, with untreated controls (represented by the blue dotted line, No inh.) set as 100%. **(h, i)** The cell number and % of dead cells (7AAD^+^) analysis in Ramos cells after a 4‐day culture period, comparing untreated (−) to treated with DARA, ISA or beriglobin control. **(j)** Cell cycle stages in Ramos cells stained with Vibrant DyeCycle in the same experimental setup as **(h, i)**. **(k)** % of cells in G1, S and G2/M phase. Statistical significance was calculated with one‐way ANOVA with the post hoc Tukey test and denoted as *P < 0.05; **P < 0.01; ***P < 0.001; ****P < 0.0001. Data are presented as mean ± SD. Non‐significance is not indicated in the figure. Data in **(g)** are from two independent experiments with *n* = 3 replicates each, with results normalised and combined. Data in **(h–k)** are representative of one experiment with *n* = 3 replicates. Independent experiments were repeated at least twice.

CD38 is a transmembrane protein characterised by a short 20‐aa N‐terminal cytoplasmic tail, a transmembrane helix and an extended extracellular domain. This ectodomain exhibits enzymatic functions capable of catalysing the conversion of nicotinamide adenine dinucleotide (NAD) into cyclic adenosine diphosphate ribose (cADPR) and ADPR,^25^ the generation of nicotinic acid adenine dinucleotide phosphate (NAADP) from NADP^26^, ^27^ and hydrolysis of NAADP and cADPR.^28^ cADPR and NAADP play pivotal roles in mobilising intracellular Ca^2+^ reservoirs,^29^, ^30^ while ADPR facilitates Ca^2+^ influx across the plasma membrane. Specifically, ADPR serves as a ligand for the transient receptor potential melastatin subfamily member 2 (TRPM2) channel, a calcium‐permeable non‐selective cation channel that can be activated by extracellular ADPR. Activation of TRPM2 leads to increased intracellular calcium levels, which are crucial for various cellular processes such as cell viability, activation and apoptosis.^31^, ^32^ These enzymatic reactions take place in an active site pocket in the CD38 ectodomain, located opposite to where DARA and ISA bind their epitopes, which recognise 28 and 23 aa, respectively (PDB entry: 1YH3) (Figure 2a and b and Supplementary figure 2).^33^, ^34^, ^35^, ^36^, ^37^, ^38^ *In vitro* studies on multiple myeloma cell lines and purified CD38 protein have shown that ISA inhibits both CD38 hydrolase and cyclase activity, while DARA only partially inhibits cyclase activity and enhances hydrolase activity.^20^, ^39^ A previous study reported that ISA leads to significant conformational changes in CD38, and these inhibit its cyclase activity through an allosteric mechanism, without obstructing the catalytic site.^36^ Given that neither mAbs directly target the active site, we investigated whether their binding induced conformational changes in the CD38 ectodomain that could potentially explain the observed differences in enzymatic inhibition. The crystal structure of the unbound CD38 ectodomain (PDB entry: 1YH3) provides a baseline view of the protein in its apo form, with an open and accessible active site (Figure 2c). This serves as a reference to understand the structural changes upon ligand binding. The NAD^+^‐bound and ADPR‐bound structures of CD38 (PDB entries: 3OFS and 8P8C, respectively) show a change in the active site conformation, reflecting different substrate‐bound forms (Figure 2d). To explore the effects of antibody binding, we analysed the structures of CD38 in complex with daratumumab (PDB entry: 7DHA)^40^ and isatuximab (PDB entry: 4CMH),^36^ which were examined without any bound substrates to focus solely on the structural changes induced by antibody binding. The CD38–DARA complex exhibited minimal alterations in the active site, while ISA binding caused significant conformational shifts (Figure 2e and f), potentially explaining the greater inhibition of enzymatic activity observed in previous studies.^20^ To substantiate these findings, we performed a cyclase assay on purified CD38 proteins. In this assay, we evaluated the impact of DARA and ISA on CD38 cyclase activity and compared it with the impact of quercetin, a flavonoid serving as a positive control because of its potent inhibitory effect on CD38 cyclase activity. The results demonstrated that ISA significantly inhibited CD38 cyclase activity, showing substantially greater inhibition than that observed with quercetin (Figure 2g). In contrast, DARA exhibited only partial inhibition of CD38 cyclase activity, which did not reach the low levels observed with quercetin and ISA, not even at higher concentrations. These findings support the hypothesis that antibody‐induced conformational changes in CD38 differentially affect enzymatic activity, with ISA inducing more pronounced conformational alterations that result in greater enzymatic inhibition. Moreover, the significant molecular weight disparity between quercetin (302.24 Da) and ISA (approximately 150 000 Da) results in notable differences in their effectiveness at equivalent molar concentrations, with ISA demonstrating much greater inhibitory potency.

### Ramos cells as a suitable model for CD38 mAb therapy in vitro studies in pBL

To investigate the impact of DARA and ISA on pBL cells, we sought to identify a suitable model for our *in vitro* analyses. We utilised a comprehensive RNA‐seq data set of 1450 cell lines (Supplementary figure 3a) and assessed *CD38* expression. Our analysis indicated that lymphoid and myeloid lineages exhibited the highest *CD38* levels (Supplementary figure 3b). Examination of these two lineages disclosed four disease subtypes with significantly higher *CD38* expression relative to the others, that is adult T‐cell leukaemia, B‐lymphoblastic leukaemia, BL and DLBCL (Supplementary figure 3c). Comparing the four subtypes revealed BL as the disease with the most consistent *CD38* expression. Given its close match with the median *CD38* expression in BL cell lines and its extensive use in our previous research, the Ramos cell line emerged as the optimal choice for our investigative model on pBL.

### ISA outcompetes DARA in killing malignant B cells and inhibiting their proliferation

To evaluate the efficacy of DARA and ISA as potential treatments for pBL, Ramos B cells were cultured for 4 days with DARA or ISA (1 mg mL^−1^). Cells untreated (−) and exposed to beriglobin (1 mg mL^−1^) were used as control. Given that DARA and ISA are injectable agents, we chose beriglobin, as it is also an injectable solution derived from pooled human plasma donations, rich in IgGs. ISA effectively reduced cell proliferation by 80% compared to 39% observed with DARA (Figure 2h). This difference appeared to stem from ISA's superior ability to both induce cell death (ISA 18% vs DARA 5%) and inhibit cell division (cells in G2/M, ISA 8% vs DARA 13% vs control groups 16%) (Figure 2i–k and Supplementary figure 4). No significant difference was observed between the untreated cells and those treated with beriglobin, as expected considering Ramos B cells, in contrast to healthy B cells, do not express FcgRII, a finding consistent with previous studies (Supplementary figure 5).^41^ Thus, beriglobin was excluded from subsequent experiments. In summary, the data indicate that ISA is a more potent agent than DARA in both killing pBL cells and inhibiting proliferation of the surviving cells.

### ISA treatment reduces intracellular Ca^2+^ response in pBL

**Figure 3:**
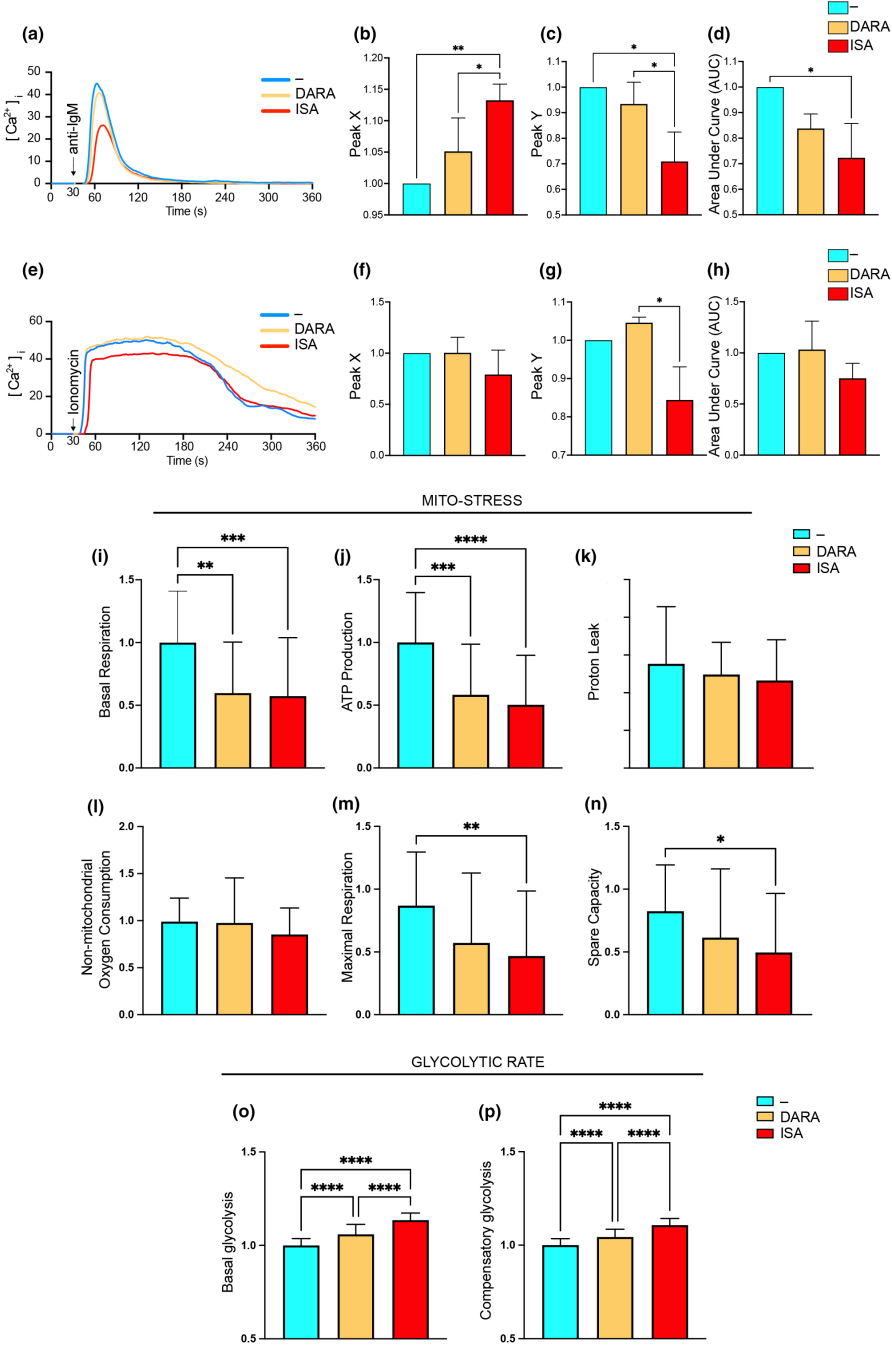
DARA and ISA impact on intracellular Ca^2+^ signalling and metabolic changes in pBL cells. **(a)** Transient rise in [Ca^2+^]_i_ over time in Ramos cells, untreated (−) or treated with DARA or ISA, following stimulation with anti‐IgM, F(ab')_2_. The curves illustrate the temporal dynamics of [Ca^2+^]_i_. **(b)** Peak X indicates the time (seconds, s) to reach maximum [Ca^2+^]_i_, **(c)** Peak Y the maximum [Ca^2+^]_i_ achieved and **(d)** the Area Under Curve (AUC) the total Ca^2+^ released. **(e)** Transient rise in [Ca^2+^]_i_ over time in Ramos cells, untreated (−) or treated with DARA or ISA, following stimulation with ionomycin. The curves illustrate the temporal dynamics of [Ca^2+^]_i_. **(f)** Peak X indicates the time (seconds, s) to reach maximum [Ca^2+^]_i_, **(g)** Peak Y the maximum [Ca^2+^]_i_ achieved and **(h)** the Area Under Curve (AUC) the total Ca^2+^ released. **(i–p)** Ramos cells, untreated (−) or treated with DARA or ISA for 1 h were assessed for metabolic changes involving mitochondrial respiration (Mito‐stress) and glycolysis (Glycolytic rate). **(i)** Basal respiration rates and **(j)** ATP production. **(k, l)** Proton leak and non‐mitochondrial oxygen consumption, respectively. The maximal respiration rate and spare respiratory capacity were depicted in **m** and **n**. Glycolytic function is assessed in **(o)** and **(p)** where basal and compensatory glycolysis levels were shown. Statistical significance was calculated with one‐way ANOVA with the post hoc Tukey test and denoted as *P < 0.05; **P < 0.01; ***P < 0.001; ****P < 0.0001. Data are presented as mean ± SD. Non‐significance is not indicated in the figure. **(a–n)** Data presented are from three independent experiments, with results normalised and combined. **(o, p)** Data presented are from two independent experiments, with results normalised and combined.

We previously demonstrated that DARA targeting CD38 disrupts BCR signalling in both normal and malignant B cells.^11^ BCR antigen binding triggers a signalling cascade, leading to the release of intracellular Ca^2+^ from the endoplasmic reticulum and the influx of extracellular Ca^2+^ via store‐operated calcium entry (SOCE) and calcium release‐activated calcium (CRAC) channels.^42^ CD38 is involved in the formation of molecules that act as secondary messengers for Ca^2+^ release, we therefore investigated the effect of DARA and ISA on Ca^2+^ release in pBL cells. Ramos cells were either untreated (−) or treated with DARA or ISA (1 mg mL^−1^ for 1 h) and labelled with Fura Red and Fluo‐3 Ca^2+^ indicator dyes. After 30 s from the start of the experiment, the cells were stimulated with an anti‐IgM F(ab')_2_. We monitored the signal curve indicative of the rise and decline of Ca^2+^ concentration in the cytosol, [Ca^2+^]_i_, for a total of 360 s. ISA‐treated cells exhibited a weaker response than those treated with DARA or left untreated (Figure 3a). We then analysed three aspects of these experiments: Peak X marks the time point of maximum [Ca^2+^]_i_; Peak Y quantifies the maximum [Ca^2+^]_i_ value; and Area Under Curve (AUC) provides measurement of the total Ca^2+^ released throughout the experiment. Cells treated with ISA exhibited slower [Ca^2+^]_i_ increase than the other groups and had the lowest peak of [Ca^2+^]_i_, as well as the lowest AUC, especially when compared to the untreated sample (Figure 3b–d). This indicates that intracellular Ca^2+^ release following IgM activation is diminished in cells treated with ISA, suggesting that it attenuates signalling pathways downstream of BCR activation. To test the specificity of CD38 inhibition in limiting BCR‐mediated Ca^2+^ flux response, another experiment was conducted, utilising ionomycin, a ionophore that promotes the influx of extracellular Ca^2+^ and the release of Ca^2+^ from internal stores.^43^ Ionomycin induced a substantial increase in [Ca^2+^]_i_ across all treatment and control group, and no significant difference was observed between the untreated group and those treated with DARA or ISA (Figure 3e–h). Nevertheless, a trend was observed in which cells treated with ISA exhibited a lower peak Y than the untreated group, and this reduction was significant when compared to the DARA‐treated cells (Figure 3g). This discrepancy might be attributed to a diminished availability of intracellular Ca^2+^, since the extracellular Ca^2+^ levels remained constant across all groups. The variance observed in the response of ISA‐treated cells, especially when intracellular Ca^2+^ sources were presumably the primary contributors, suggests that ISA might affect intracellular Ca^2+^ availability or regulation.

### Metabolic shift in pBL cells following anti‐CD38 mAb treatment

Intracellular Ca^2+^ plays a pivotal role in cellular metabolism, acting as a crucial secondary messenger that orchestrates a multitude of processes, from enzyme activities to mitochondrial function.^44^ Using the Seahorse method, we sought to measure cellular respiration and energy production in real time and explore the intricate interplay between anti‐CD38 treatments and cellular metabolic pathways. The Mito‐Stress test is designed to assess the respiratory capacity and adaptability of cells or mitochondria in the face of external challenges. Our observations indicated that the oxygen consumption under resting conditions (basal respiration) and the ATP production were lower for the anti‐CD38‐treated groups (1 mg mL^−1^ for 1 h), compared with the untreated one, with ISA showing a more pronounced effect (Figure 3i and j). Proton leak and non‐mitochondrial oxygen consumption remained relatively uniform across all groups (Figure 3k and l), while the maximal respiration rate and spare respiratory capacity was reduced in the ISA‐treated cells, indicating a constrained respiratory flexibility compared with the untreated control (Figure 3m and n). We then performed a glycolytic rate test and found differences in basal and compensatory glycolysis. The cells treated with ISA and DARA exhibited rates significantly higher than the untreated control, with ISA treatment resulting in the highest rate (Figure 3o and p). At the same time, we performed Mito‐Stress and glycolytic rate tests with the cells exposed to the treatment for an additional h, that is 2 h in total (Supplementary figure 6). The results were similar to those recorded after 1‐h treatment, with the difference of DARA treatment seemingly catching up with ISA for both tests and ISA showing a lesser impact to the metabolic shift. These findings underscore a distinctive metabolic reprogramming under compromised mitochondrial respiration in malignant B cells prompted by anti‐CD38 therapy, with ISA demonstrating a faster influence on these metabolic changes compared with DARA.

### ISA superiority in inhibiting IgM:CD19 Interaction

**Figure 4:**
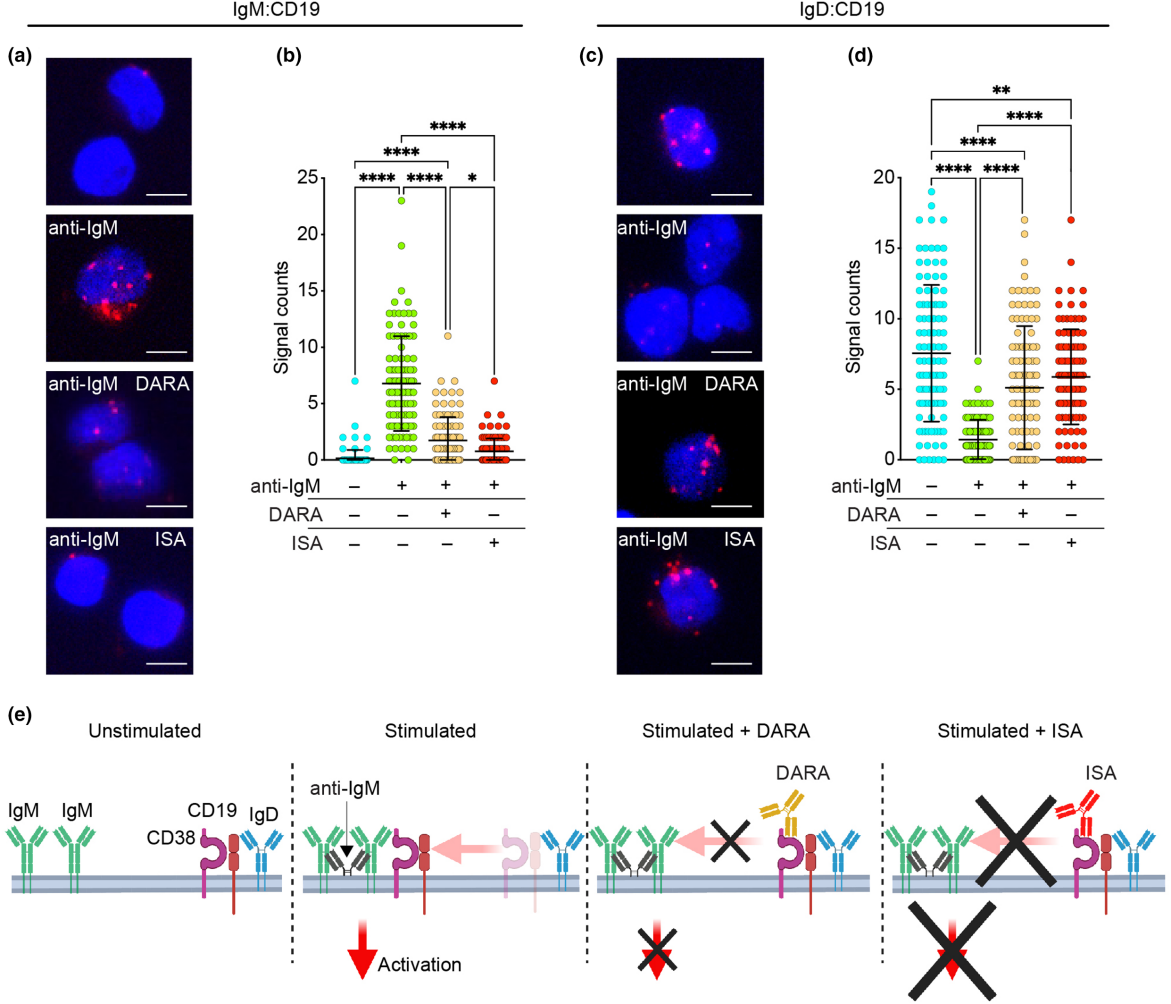
Comparative efficacy of DARA and ISA on modulating IgM:CD19 and IgD:CD19 interactions. **(a)** Fab‐PLA study of the proximity of IgM to CD19 on Ramos cells unstimulated or 5‐min anti‐IgM–stimulated without and with exposure to DARA or ISA (top to bottom). PLA signals are shown in red and nuclei in blue. Scale bar, 5 μm. **(b)** Scatter dot plot represents the mean of PLA signals for IgM:CD19 interaction (signal counts). **(c)** Fab‐PLA study of the proximity of IgD to CD19 on Ramos cells unstimulated or 5‐min anti‐IgM–stimulated without and with exposure to DARA or ISA (top to bottom). PLA signals are shown in red and nuclei in blue. Scale bar, 5 μm. **(d)** Scatter dot plot represents the mean of PLA signals for IgD:CD19 interaction (signal counts). Shown are representative microscope images **(a, c)**. Statistical significance in this figure was calculated with one‐way ANOVA with the post hoc Tukey test and denoted as *P < 0.05; **P < 0.01; ****P < 0.0001. Non‐significance is not indicated in the figure. Independent experiments were repeated twice. In these graphs, every data point is one cell; error bars show mean ± SD. **(e)** Schematic representation of IgM and IgD interactions with CD19 and CD38 upon BCR activation, detailing the distinct inhibitory impacts of DARA and ISA.

Upon maturation, B cells simultaneously express two BCR isotypes, IgM and IgD, each organised into distinct class‐specific nanoclusters on the cell surface. In the absence of an antigen, B cells typically exhibit CD19 in proximity to the IgD‐BCR. During early B‐cell activation, CD19 promotes BCR‐antigen microcluster formation.^45^ We have previously shown that antigen binding to the IgM‐BCR promotes the association of IgM with CD19 and that DARA inhibits this association.^11^, ^18^ These discoveries led us to investigate whether ISA could similarly affect this interaction by using the Fab‐based proximity ligation assay (Fab‐PLA).^46^ Ramos cells were either left unstimulated or stimulated for 5 min with anti‐IgM. Prior to stimulation, the cells were treated with either DARA or ISA (1 mg mL^−1^) for 1 h or left untreated. Notably, DARA and ISA treatments did not induce any change in IgM, IgD or CD19 surface expression (Supplementary figure 7). Unstimulated cells displayed low IgM:CD19 interaction signals, which were significantly increased upon stimulation (Figure 4a and b). Cells exposed to anti‐CD38 mAbs showed reduced IgM:CD19 interaction. ISA‐treated cells demonstrated signal counts similar to unstimulated cells and significantly lower than those observed in DARA‐treated cells. Extending our analysis to the IgD:CD19 interaction, unstimulated Ramos cells displayed a close association between these surface proteins, which diminished upon anti‐IgM stimulation (Figure 4c and d). Both DARA and ISA treatments were effective in preserving the IgD:CD19 interaction, with ISA‐treated cells more closely resembling the unstimulated state than DARA‐treated cells. In summary, ISA exhibited a more marked inhibitory effect on IgM:CD19 proximity than DARA and better preserved the IgD:CD19 association. Coupled with the findings from the Ca^2+^ flux experiments, these differences may account for the distinct impacts on downstream signalling cascades initiated by the IgM‐BCR, as depicted schematically in Figure 4e.

### Differential effects of anti‐CD38 mAbs on PI3K pathway

**Figure 5:**
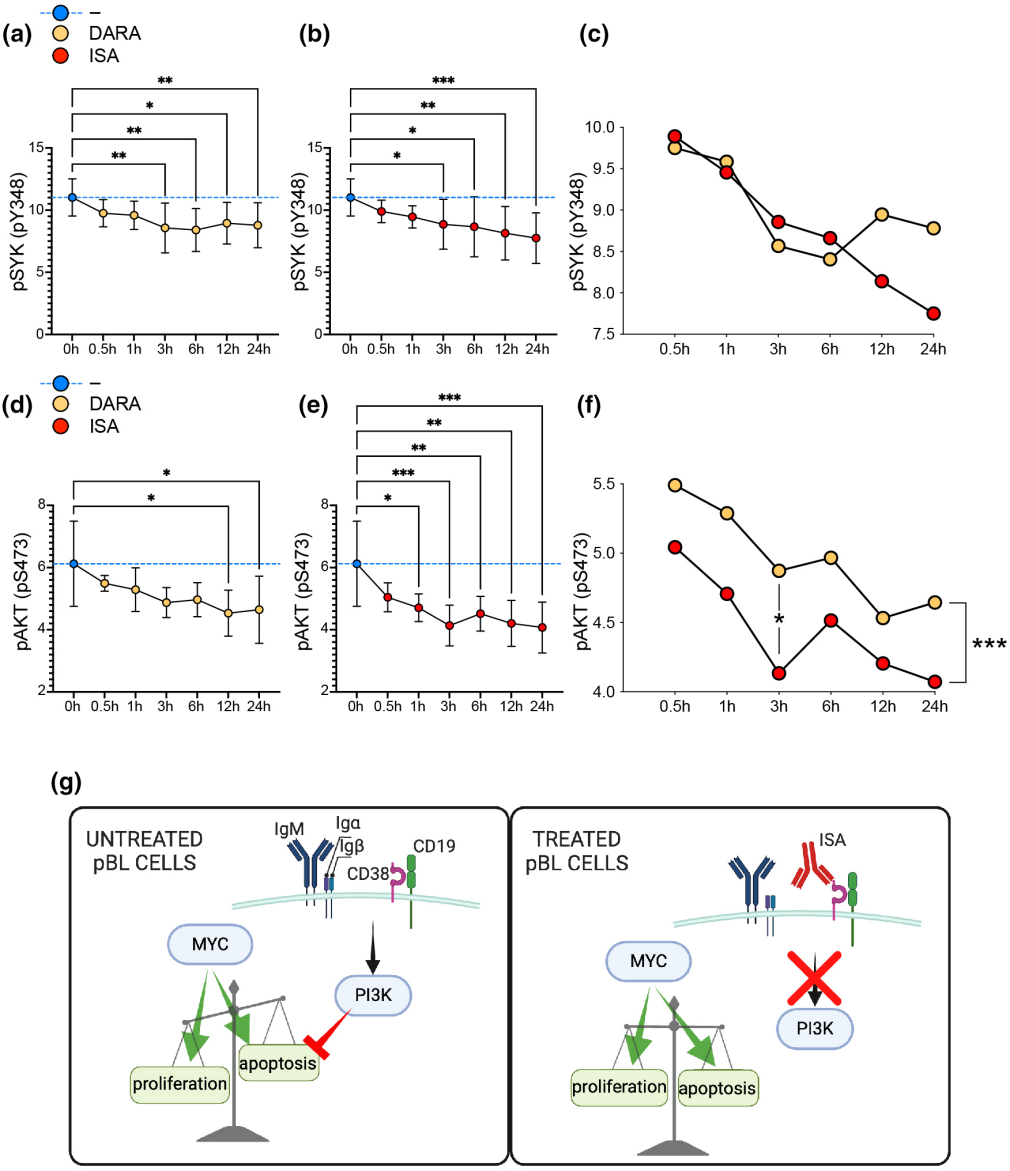
Anti‐CD38 mAbs impair PI3K pathway signalling in pBL cells. **(a, b)** Time‐course analysis of SYK phosphorylation levels post anti‐CD38 mAb treatment over 24 h. **(c)** Comparative kinetic analysis of SYK dephosphorylation between DARA and ISA‐treated cells. **(d, e)** The phosphorylation status of AKT over a 24‐h period post anti‐CD38 mAb treatment. **(f)** Differential analysis of pAKT level kinetics between DARA and ISA treatments. Statistical significance was assessed using one‐way ANOVA with the post hoc Tukey test comparing untreated vs treated, indicated by: *P < 0.05; **P < 0.01; ***P < 0.001. In **(c)** and **(f)**, the statistical significance was assessed using the unpaired *t*‐test for the comparison between DARA vs ISA in each time point and the paired *t*‐test for the comparison of the whole kinetic, and indicated by *P < 0.05; ***P < 0.001. Data are presented as mean ± SD. Non‐significance is not denoted. Data are representative of two independent experiments. All results were normalised and merged for consistent interpretation. **(g)** Schematic representation of the proposed effect of anti‐CD38 mAb (ISA) treatment on MYC/PI3K pathways and consequences on the proliferation/apoptosis balance.

Our findings thus far led us to examine the signalling pathways implicated in the decreased survival of pBL cells following anti‐CD38 mAb treatment. Previous studies have established the central role of antigen‐independent BCR signalling, resembling ‘tonic’ signalling, in sustaining the survival of malignant B cells in pBL, a process largely driven by the PI3K pathway.^17^ CD19 is a major player in the PI3K signalling pathway, and it is essential for effective activation of AKT, a key kinase downstream the PI3K signalling cascade following BCR engagement.^47^ In addition, SYK has been identified as a key player in tonic signalling, and it is known to be upstream the PI3K pathway.^48^, ^49^, ^50^, ^51^ In B‐CLL, DARA interferes with BCR signalling by downregulating the phosphorylation of several kinases including SYK and AKT.^52^ Similarly, DARA modulates BCR and AKT‐associated signalling in Waldenström macroglobulinaemia.^53^ To better grasp the mechanisms of anti‐CD38 mAbs effects on pBL, we performed a 24‐h time‐course analysis of SYK pY348 phosphorylation (pSYK) by flow cytometry and compared pSYK levels of cells untreated and treated with either DARA or ISA. This revealed a significant reduction in pSYK levels starting at 3‐h post‐mAbs exposure, with a decrease throughout the 24‐h period (Figure 5a and b). Relative to the untreated cells (0 h), the cells treated with DARA presented slightly more pronounced dephosphorylation of SYK after 3 and 6 h than ISA. Conversely, ISA‐treated cells displayed a steady decline in pSYK at the 12‐ and 24‐h time points, while pSYK levels plateaued in DARA‐treated cells. Direct comparison between DARA and ISA highlighted distinct kinetic patterns, but with no significant differences (Figure 5c). We then examined phosphorylation of AKT pS347 (pAKT). The results revealed that the cells subjected to DARA showed a significant reduction in pAKT levels, but only after 12 and 24 h of treatment. In contrast, the ISA‐treated cells showed an early decrease in pAKT levels already at 1‐h post‐treatment, which persisted over the 24 h (Figure 5d and e). This led to a marked divergence between the kinetics of DARA and ISA treatments, with the latter showing consistently lower pAKT levels, with the most pronounced difference after 3 h (Figure 5f). This significant reduction in pAKT levels in ISA‐treated Ramos cells was confirmed by immunoblotting analysis (Supplementary figure 8). However, the data indicated that ISA resulted in lower pAKT levels compared with DARA at the 1‐h time point, while the difference was less pronounced at 3 h.

## Discussion

In the context of pBL, the PI3K pathway plays a critical role by providing a pro‐survival counterbalance to the apoptotic side of the oncogene MYC. In cervical cancer, CD38 enhances phosphorylation of PI3K, AKT and mechanistic target of rapamycin (mTOR), all crucial for cell survival and proliferation, mediated by its NADase activity impacting NAD levels and cellular metabolism.^54^ Furthermore, recent research has identified a regulatory axis comprising CD38, NAADP and the transcription factor EB (TFEB), the latter being repressed by MYC and activated by the CD38/NAADP pathway.^55^ This interaction is particularly significant as TFEB acts as a tumor suppressor in acute myeloid leukaemia by promoting myeloid differentiation and cell death, thereby emphasising the critical nature of the CD38–MYC relationship and introducing additional complexity.^56^ The positive correlation between CD38 and MYC expressions in pBL patient samples observed in our analysis further justifies targeting CD38 given the pivotal role of MYC in the oncogenesis of this disease. We previously observed that targeting CD38 with DARA significantly reduced proliferation and survival in both normal B cells and in pBL cell lines.^11^ A subsequent study by others demonstrated that DARA also impairs B‐cell differentiation into plasma cells, reduces antibody production and further confirmed the inhibition of B‐cell proliferation.^57^ Our study sheds light on the intricate roles of CD38 and the PI3K pathway in the pathophysiology of pBL and their potential as therapeutic targets (Figure 5g). Here, we have shown that anti‐CD38 mAbs have profound effects on the signalling mechanisms essential for pBL cell survival and proliferation. The early and sustained decrease in pAKT levels upon ISA treatment, along with the significant reduction in pSYK, suggests that ISA disrupts the PI3K pathway more effectively than DARA, which may explain the superior efficacy of ISA in inhibiting proliferation and inducing cell death. This disruption is significant, given the central role of the PI3K pathway in supporting the survival of malignant pBL cells and its role in chemotherapy resistance, potentially offering a therapeutic advantage. Additionally, ISA induces a rapid and persistent metabolic reprogramming in pBL cells resembling the Warburg effect,^58^ in which increased glycolysis compensates for decreased mitochondrial respiration, albeit ineffectively, as ISA efficiently inhibits proliferation and induces cell death, in contrast to the more gradual and mild effects induced by DARA. This metabolic reprogramming may provide a window of therapeutic opportunity, as inhibitors targeting glycolytic enzymes or glucose transporters could be used in combination with ISA to exploit the vulnerability of certain cancer cells, potentially leading to better clinical outcomes.^59^, ^60^ Moreover, after BCR engagement, ISA showed a superior efficacy compared with DARA in preventing IgM:CD19 interaction and Ca^2+^ signalling, the latter being a process where DARA was unsuccessful. Nevertheless, caution is warranted in directly linking the different outcomes between DARA and ISA treatments to the enzymatic activity of CD38. In this study, we show the superiority of ISA in inhibiting the cyclase activity of CD38 compared with DARA. DARA and ISA specifically target extracellular type II CD38, which has limited cADPR‐producing cyclase activity and primarily metabolises NAD to ADPR and nicotinamide.^61^ The major producer of cADPR, cytosolic type III CD38, which significantly influences Ca^2+^ release from intracellular stores, is not accessible to these mAbs. Therefore, further studies with direct CD38 inhibitors are essential to clarify the specific effects of type II CD38 on cellular signalling and to validate the enzymatic impact on cellular responses. However, ADPR produced extracellularly by CD38 through NAD degradation activates TRPM2, which also plays a role in Ca^2+^ signalling, linked to the survival, proliferation and metabolic status of cancer cells.^62^ Furthermore, ADPR is processed by CD203a to adenosine monophosphate (AMP) and ultimately converted to adenosine by CD73, thereby inducing an immunosuppressive tumor microenvironment.^62^ The ability of ISA to inhibit both the cyclase and hydrolase activities of CD38, in contrast to the more limited effect of DARA on only the cyclase activity, highlights its critical role in targeting CD38's extracellular enzymatic functions. Importantly, DARA induces significant redistribution of CD38 on the myeloma cell membrane, resulting in the release of CD38‐rich microvesicles, whereas ISA primarily remains on the cell surface or undergoes partial internalisation.^63^ These differences in antigen–antibody dynamics may further explain the superior efficacy of ISA. Our observations are in line with recent developments in CD38‐targeted therapies, such as the findings on the novel humanised anti‐CD38 mAb FTL004, which has shown enhanced pro‐apoptotic activity with minimal red blood cells (RBCs) binding.^64^ In this study, ISA exhibited a superior ability to induce direct apoptosis in various B‐cell lines, including Ramos, after 24‐h treatment compared with DARA, with a significant reduced interaction with RBCs suggesting potential for increased efficacy and safety in treatment protocols. Moreover, recent findings have shown that IgE mAbs targeting CD38 demonstrate significant ADCC and ADCP effects against MM cells.^65^ Interestingly, Ramos cells do not express Fcγ receptors but do express low levels of CD23, the low‐affinity Fcε receptor (FcεRII).^66^ This receptor is known to bind IgE and could potentially be utilised to induce the ‘scorpion effect’ or ‘Kurlander effect’, which occurs when an antibody binds both the target protein with its Fab region and an Fc receptor with its Fc region simultaneously, enhancing cytotoxicity.^67^ Finally, the inhibitory interaction of CD38 on the stimulator of interferon genes (STING) shown in MM, although less defined, adds a layer of complexity to possible immune modulation and therapeutic responses also in pBL, influencing tumor immune evasion and treatment effectiveness.^68^ In conclusion, our results suggest that targeting CD38 with mAbs, in particular ISA, could improve pBL treatment for children not cured by current therapies.

## Methods

### Online data set analyses

The gene expression data comprising the collection of different cell lines used in this manuscript were obtained from publicly available data sets at https://depmap.org. The gene expression data comprising paediatric lymphomas were collected from published studies.^22^, ^24^ These data are available in the National Center for Biotechnology Information (NCBI) Gene Expression Omnibus (GEO) database (https://www.ncbi.nlm.nih.gov/geo/; accession numbers GSE10172 and GSE64905). Gene expression data from RNA‐seq and microarrays were analysed using Qlucore Omics Explorer 3.9.9 (Qlucore AB, Lund, Sweden).

### Crystal structure visualisation

The crystal structure for the extracellular domain of human CD38 in its unbound form (PDB entry: 1YH3) and bound to ADPR (PDB entry: 8P8C), NAD^+^ (PDB entry: 3OFS), DARA (PDB entry: 7DHA) and ISA (PDB entry: 4CMH) were obtained from the RCSB Protein Data Bank (PDB) (https://www.rcsb.org). ChimeraX v1.6.1 (https://www.cgl.ucsf.edu/chimerax) and Biorender (https://www.biorender.com) were used to generate images.

### CD38‐cyclase assay

The CD38 cyclase assay was conducted using the CD38 Inhibitor Screening Assay Kit (BPS Bioscience, San Diego, California, USA) according to the manufacturer's protocol. The assay included purified CD38 protein to assess cyclase activity by measuring the production of nicotinamide from the substrate nicotinamide guanine dinucleotide (NGD^+^). The fluorescence of the resulting product was quantified using a Spectramax ID5 plate reader (Molecular Devices, San Jose, California, USA), with an excitation wavelength of 300 nm and an emission wavelength of 410 nm. The efficacy of CD38 inhibition was evaluated by comparing the titrations of the inhibitors daratumumab (DARA, trade name Darzalex®, human IgG1κ, HuMax®‐CD38, Genmab, Copenhagen, Denmark, and Janssen, Beerse, Belgium) and isatuximab (ISA, trade name Sarclisa®, chimeric‐humanised IgG1κ, anti‐CD38, Sanofi, Paris, France) against quercetin (BPS Bioscience, San Diego, California, USA) at concentrations of 0.1, 1, 10 and 100 μg mL^−1^, with quercetin serving as the positive control. The background values obtained from the wells containing only cyclase buffer were subtracted from all sample readings. Subsequently, the results were then normalised to the negative control (no inhibitor), which was set as 100% CD38 cyclase activity.

### Proliferation, apoptosis and cell cycle

Ramos cells (ATCC, Manassas, Virginia, USA) were cultured in 96‐well plates in complete medium consisting of IMDM supplemented with GlutaMAX 1% (Life Technologies, Waltham, Massachusetts, USA), Penicillin/Streptomycin 10 mM (Life Technologies, Waltham, Massachusetts, USA), FBS 10% (Gibco, Waltham, Massachusetts, USA) and 2‐β‐mercaptoethanol (50 μM) at an initial concentration of 10^4^ cells/well without or with 1 μg mL^−1^ Beriglobin (Human IgG control, CSL Behring, King of Prussia, Pennsylvania, USA), DARA or ISA, 37°C, 5% CO_2_. After 4 days, cells were harvested and incubated with Vybrant® DyeCycle™ Violet Stain (Invitrogen, Carlsbad, California, USA) for 30 min to identify cell cycle stages and 7AAD (BD Biosciences, San Jose, California, USA) for 5 min to identify late apoptotic cells. The stainings were performed without washing steps according to the manufacturer's protocols, which allowed for accurate determination of cell proliferation by flow cytometry acquisition with a BD FACS Lyric (BD Biosciences, San Jose, California, USA) and subsequent data analysis by the FlowJo software (BD Biosciences, San Jose, California, USA).

### Intracellular Ca^2+^ flux

Changes in the concentration of intracellular Ca^2+^ [Ca^2+^]_i_ was measured by flow cytometry. Ramos cells (10^7^) were incubated in 1 mL of complete medium, composed as for the previous experiments, in the absence or presence of ISA (1 μg mL^−1^) or DARA (1 μg mL^−1^) for 1 h, 37°C, 5% CO_2_. The cells were then washed with 10 mL of Krebs–Ringer phosphate buffer (KRG; 120 mM NaCl, 5 mM KCl, 1.7 mM KH_2_PO_4_, 8.3 mM NaH_2_PO_4_, 1.2 mM MgSO_4_, 10 mM glucose, and 1 mM CaCl_2_ in dH_2_O, pH 7.3) supplemented with FBS (1%) by centrifugation (300 × *g*, 22°C, 10 min). After aspiration of the supernatant, the cells were resuspended in 1 mL KRG with FBS (1%) and stained with Fluo‐3‐acetoxymethyl (AM) (4 μg mL^−1^; # F1242 Invitrogen, Carlsbad, California, USA) and FuraRed‐AM (10 μg mL^−1^; # F3020, Invitrogen, Carlsbad, California, USA) for 30 min at 37°C. Thereafter, the cells were washed twice with ice‐cold 10 mL KRG with FBS (1%) by centrifugation (300 × *g*, 4°C, 10 min) before resuspended in 0.5 mL KRG with FBS (1%) for a concentration 2 × 10^7^ cells mL^−1^ and kept on ice in darkness. For evaluation of the transient rise in [Ca^2+^]_i_ 60 μL stained cells were diluted in 510 μL KRG and equilibrated at 37°C for 4 min, prior addition of 30 μL stimulus (ionomycin, Sigma‐Aldrich, Burlington, Massachusetts, USA, or anti‐IgM, F(ab')_2_, Jackson ImmunoResearch Laboratories, West Grove, Pennsylvania, USA) and measurement of Fluo‐3/Fura‐Red continuously over time on a BD FACS Lyric (BD Biosciences, San Jose, California, USA). The data were analysed by the FlowJo software (BD Biosciences, San Jose, California, USA) by calculating the ratio between Fluo‐3 and FuraRed to reflect the relative increase in [Ca^2+^]_i_ over time.

### Measurement of oxygen consumption rate and glycolytic rate using Seahorse

The oxygen consumption rate (OCR) was measured with the Mito‐stress test, and the basal and compensatory glycolysis were measured with the glycolytic rate assay test using Seahorse XFe96 metabolic flux analyser (Agilent Technologies, Santa Clara, California, USA). Cells (1.5 × 10^6^ cells mL^−1^) were either untreated or treated with anti‐CD38 mAbs ISA (1 μg mL^−1^) or DARA (1 μg mL^−1^). The treatments were done in Seahorse cell assay medium (unbuffered DMEM medium from Agilent Technologies supplemented with 10 mM glucose and 2 mM glutamine, pH 7.4). One set of cells were treated with mAbs just prior addition to Seahorse cell plates, and another set of cells were pre‐treated with mAbs for 1 h at 37°C and 5% CO_2_. After the treatments, the cells were plated at a concentration of 7.6–8 × 10^4^ cells/well in seahorse cell plates that were coated with Cell Tak (Corning Inc., Corning, New York, USA) according to the manufacturer's instructions. The treated cells were then allowed to adhere for 30 min at 37°C in a non‐CO_2_ incubator. Thereafter, 130 μL of Seahorse cell assay medium was added, and the cells were incubated for another 30 min in a non‐CO_2_ incubator. In total, the cells were treated with mAbs for 1 and 2 h before performing the Seahorse metabolic assay tests. The calibrator plate was equilibrated overnight in a non‐CO_2_ incubator at 37°C in a non‐CO_2_ incubator. Ones the probe was calibrated, the calibrator plate was replaced by the cell plate. Cellular OCR was measured at basal level and after sequential injection of mitochondrial respiratory chain inhibitors: 1 μM oligomycin, 1 μM FCCP, 1 μM antimycin plus 1 μM rotenone (Sigma‐Aldrich, Burlington, Massachusetts, USA). For glycolytic rate assay, the sequential injections were with 0.5 μM antimycin plus 0.5 μM rotenone and 50 mM 2‐deoxyglucose (Sigma‐Aldrich, Burlington, Massachusetts, USA).

### Proximity ligation assay

Proximity ligation assays (PLAs) experiments were performed as previously described.^46^ To generate PLA‐probes against specific targets, the following unlabelled antibodies were used: anti‐IgM (SA‐DA4, Southern Biotech, Birmingham, Alabama, USA), anti‐IgD (6D5, Bio‐Rad AbD Serotec GmbH, Neuried, Germany) and anti‐CD19 (HIB19, Biolegend, San Diego, California, USA). To generate Fab‐fragments the buffer of the antibodies was exchanged to PBS using a Microcon‐30 kD centrifugal filter unit (MRCF0R030; Merck Millipore, Burlington, Massachusetts, USA). All Fab fragments were prepared with Pierce Fab Micro preparation kit (Thermo Fisher Scientific, Waltham, Massachusetts, USA) using immobilised papain. In brief, after desalting (Zeba spin desalting columns; Thermo Fisher Scientific, Waltham, Massachusetts, USA), all Fab‐fragments were coupled with PLA Probemaker Plus or Minus oligo‐nucleotides (Sigma‐Aldrich, Burlington, Massachusetts, USA) to generate PLA‐probes. Ramos B cells were settled on polytetrafluoroethylene (PTFE) slides (Thermo Fisher Scientific, Waltham, Massachusetts, USA) for 30 min at 37°C and incubated without or with 1 μg mL^−1^ DARA, or ISA, as described above. Ramos B cells were then stimulated for 5 min with 10 μg mL^−1^ anti‐IgM or left unstimulated and fixed for 20 min with 4% paraformaldehyde. After extensive washings, the slides were blocked for 30 min with blocking buffer (25 μg mL^−1^ sonicated salmon sperm DNA, 250 μg mL^−1^ BSA, 1 M glycine). PLA was performed with Duolink *In Situ* Orange (Sigma‐Aldrich Burlington, Massachusetts, USA). Samples were directly mounted on slides with DAPI Fluoromount‐G (Southern Biotech, Birmingham, Alabama, USA) to visualise the PLA signals in relationship to the nuclei. Microscopy images were acquired with a Leica DMi8 microscope (Leica‐microsystems, Wetzlar, Germany). For each experiment, a minimum of 100 Ramos B cells were analysed with CellProfiler‐3.0.0 (https://cellprofiler.org/).

### Immunophenotyping by flow cytometry

Ramos cells and/or peripheral blood mononuclear cells (PBMCs) were stained with the following directly labelled antibodies (all from BioLegend, San Diego, California, USA): anti‐human IgM (clone MHM‐88, fluorochrome AF647, dilution 1:100), IgD (IA6‐2, BV421, 1:100), CD19 (HIB19, PE‐Cy7, 1:100) and CD32B/C (S18005H, AF700, 1:100). The data were acquired using a FACSLyric™ (BD Biosciences, San Jose, California, USA) flow cytometer and analysed using the FlowJo software version 10 (TreeStar Inc., Ashland, Oregon, USA). PBMCs were obtained from anonymised buffy coats. As per Swedish law 2003: 460, paragraphs 4 and 13, no personal information or identity was recorded, and therefore, no written consent or approval by the Human Research Ethics Committee was required.

### Phosphoflow

The phosphorylation of SYK and AKT was analysed using a modified version of the BD PhosFlow™ Protocol for Human PBMCs. Ramos cells were incubated in complete medium without or with DARA and ISA at 1 μg mL^−1^. For the 24‐h time point, DARA and ISA were added from the beginning. For the remanent time points, DARA and ISA were added at 12, 6, 3, 1 and 0.5 h from the end of the experiment. The samples were then fixed with BD Cytofix™ Fixation Buffer (BD Biosciences, San Jose, California, USA) for 10 min at 37°C, washed twice and resuspended in Phosflow™ Perm/Wash Buffer I (BD Biosciences, San Jose, California, USA) containing mouse serum as blocking agent. Afte 15 min, the samples were stained for pSYK (pY348, moch1ct, PE, 1:100, eBioscience, San Diego, California, USA) and pAKT (pS473, M89‐61, AF647, 1:100, BD Biosciences, San Jose, California, USA) overnight at room temperature. Samples were washed and resuspended in Phosflow™ Perm/Wash Buffer I for flow cytometric analysis.

### Immunoblot

Ramos cells were treated in complete medium with or without DARA or ISA (1 μg mL^−1^) for 1 h or for 3 h. Cells were then collected and lysed on ice for 30 min in RIPA buffer supplemented with protease and phosphatase inhibitors (Halt, Thermo Fisher Scientific, Waltham, Massachusetts, USA). Protein concentrations were measured using a Qubit 3.0 fluorometer (Life Technologies, Waltham, Massachusetts, USA). Equal amounts of protein lysates were separated by SDS‐PAGE on 10% mini precast gels (7Bioscience GmbH, Neuenburg am Rhein, Germany) and transferred to PVDF membranes (GE Healthcare, Chicago, Illinois, USA). Membranes were blocked with 5% BSA in PBS containing 0.1% Tween‐20 and incubated overnight with an antibody against pAKT (pS473, D9E, Cell Signaling Technology, Danvers, Massachusetts, USA). A horseradish peroxidase (HRP)‐conjugated goat anti‐rabbit secondary antibody was applied, and detection was performed using ECL chemiluminescent substrate (Bio‐Rad, Hercules, California, USA). The membranes were stripped with NuPAGE stripping buffer for 15 min, washed with PBS‐Tween, re‐blocked and then subjected to CD38 (EPR4106, Abcam, Cambridge, UK) analysis as described above. Immunoblot analysis was performed using the ImageStudio software (Li‐Cor, Lincoln, Nebraska, USA).

### Statistical analysis

Figure captions indicate the biological replicates and statistical approaches used for each experiment. Sample size was not predetermined using statistical methods. Data distribution was assumed to be normal, and no data exclusion was performed. Statistical analyses of data from on online datasets were performed using Qlucore Omics Explorer 3.9.9 (Qlucore AB, Lund, Sweden). Statistical analyses form the other experiments were performed with Graph‐Pad Prism version 10.3.0 (GraphPad Software, La Jolla, California, USA). Levels of statistical significance are indicated by asterisks: *P < 0.05; **P < 0.01; ***P < 0.001; ****P < 0.0001. Non‐statistically significant differences are not included in the figures.

## Author contributions

**Kathrin Kläsener:** Conceptualization; data curation; formal analysis; investigation; methodology; validation; writing – review and editing. **Nadja Herrmann:** Conceptualization; data curation; formal analysis; methodology; writing – review and editing. **Liliana Håversen:** Data curation; formal analysis; investigation; methodology; software; validation; visualization; writing – review and editing. **Timothy Sundell:** Conceptualization; data curation; formal analysis; methodology; software; visualization; writing – review and editing. **Martina Sundqvist:** Conceptualization; data curation; formal analysis; funding acquisition; investigation; methodology; software; validation; visualization; writing – review and editing. **Christina Lundqvist:** Data curation; formal analysis; methodology; software; validation; visualization; writing – review and editing. **Paul T Manna:** Conceptualization; investigation; validation; writing – review and editing. **Charlotte A Jonsson:** Data curation; formal analysis; methodology; writing – review and editing. **Marcella Visentini:** Investigation; validation; writing – review and editing. **Diana Ljung Sass:** Validation; writing – review and editing. **Sarah McGrath:** Investigation; validation; writing – review and editing. **Kristoffer Grimstad:** Data curation; formal analysis; methodology; visualization; writing – review and editing. **Alaitz Aranburu:** Investigation; validation; writing – review and editing. **Karin Mellgren:** Investigation; validation; writing – review and editing. **Linda Fogelstrand:** Conceptualization; investigation; validation; writing – review and editing. **Huamei Forsman:** Investigation; validation; writing – review and editing. **Olov Ekwall:** Investigation; validation; writing – review and editing. **Jan Borén:** Investigation; validation; writing – review and editing. **Inger Gjertsson:** Investigation; resources; validation; writing – review and editing. **Michael Reth:** Investigation; methodology; resources; supervision; validation; writing – review and editing. **Inga‐Lill Mårtensson:** Conceptualization; funding acquisition; investigation; resources; validation; writing – review and editing. **Alessandro Camponeschi:** Conceptualization; data curation; formal analysis; funding acquisition; investigation; methodology; project administration; resources; software; supervision; validation; visualization; writing – original draft; writing – review and editing.

## Conflict of interest

The authors declare that the research was conducted in the absence of any commercial or financial relationships that could be considered as a potential conflict of interest. Cartoon figures were created with Biorender.com.

## Supporting information


Supplementary figure 1
Supplementary figure 2
Supplementary figure 3
Supplementary figure 4
Supplementary figure 5
Supplementary figure 6
Supplementary figure 7
Supplementary figure 8

## Data Availability

All raw and processed data of experiments included in this study are available from the corresponding authors upon reasonable request.
