# Supplementary material for: Targeting CD38 with monoclonal antibodies disrupts key survival pathways in paediatric Burkitt's lymphoma malignant B cells
Source: Clin Transl Immunology. 2024 Oct 3;13(10):e70011. doi: 10.1002/cti2.70011 (PMC11447455; doi:10.1002/cti2.70011)
Supplement: Supplementary file 1 — Supplementary figure 1 Supplementary figure 2 Supplementary figure 3 Supplementary figure 4 Supplementary figure 5 Supplementary figure 6 Supplementary figure 7 Supplementary figure 8 [file CTI2-13-e70011-s001.pdf]

Supplementary Figure 1

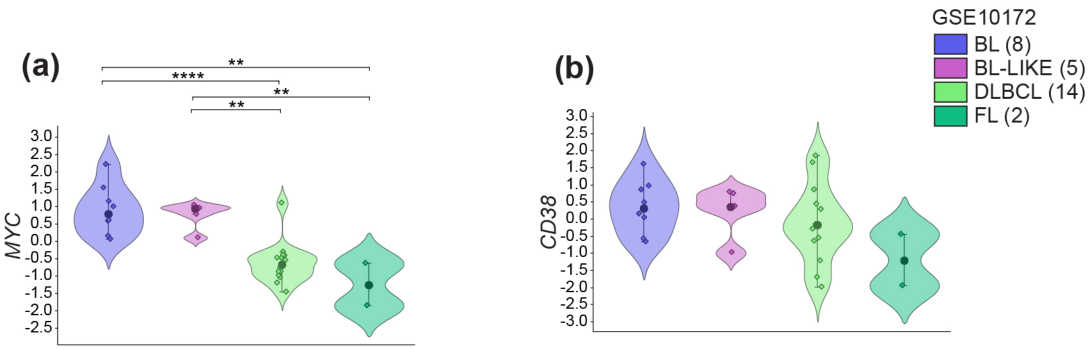

Supplementary Figure 1: CD38 and MYC expression analysis of pediatric lymphoma samples.
(a) and **(b)** show MYC and CD38 gene expression in the pediatric samples contained in GSE10172 dataset. Here the significance levels were calculated with the one-way ANOVA with post-hoc Tukey test and denoted as $^{**}P < 0.01$; $^{****}P < 0.0001$. Non-significance is not indicated in the figure.

Supplementary Figure 2

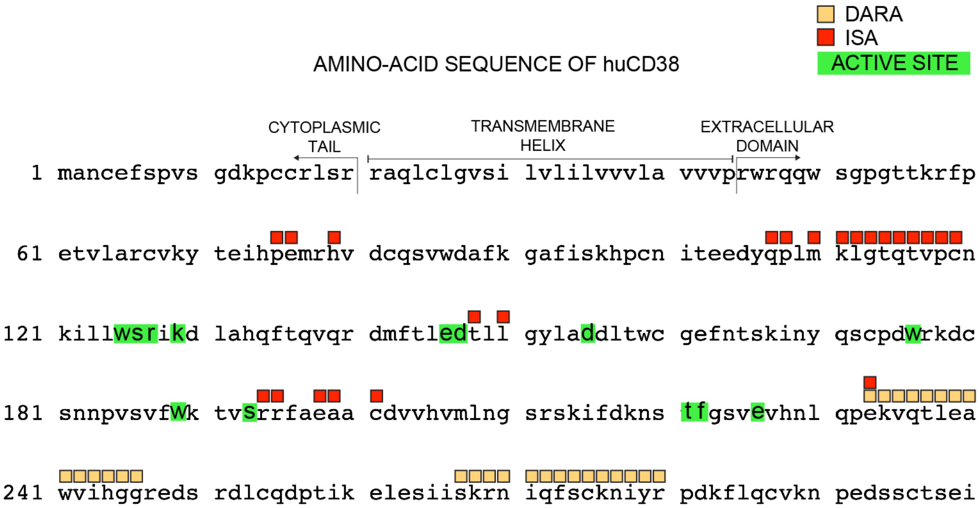

Supplementary Figure 2: Aa sequence of human CD38. Depicts the aa sequence of human CD38. The sequences of the epitopes recognized by DARA and ISA and that of the active site of the enzyme are highlighted.

Supplementary Figure 3

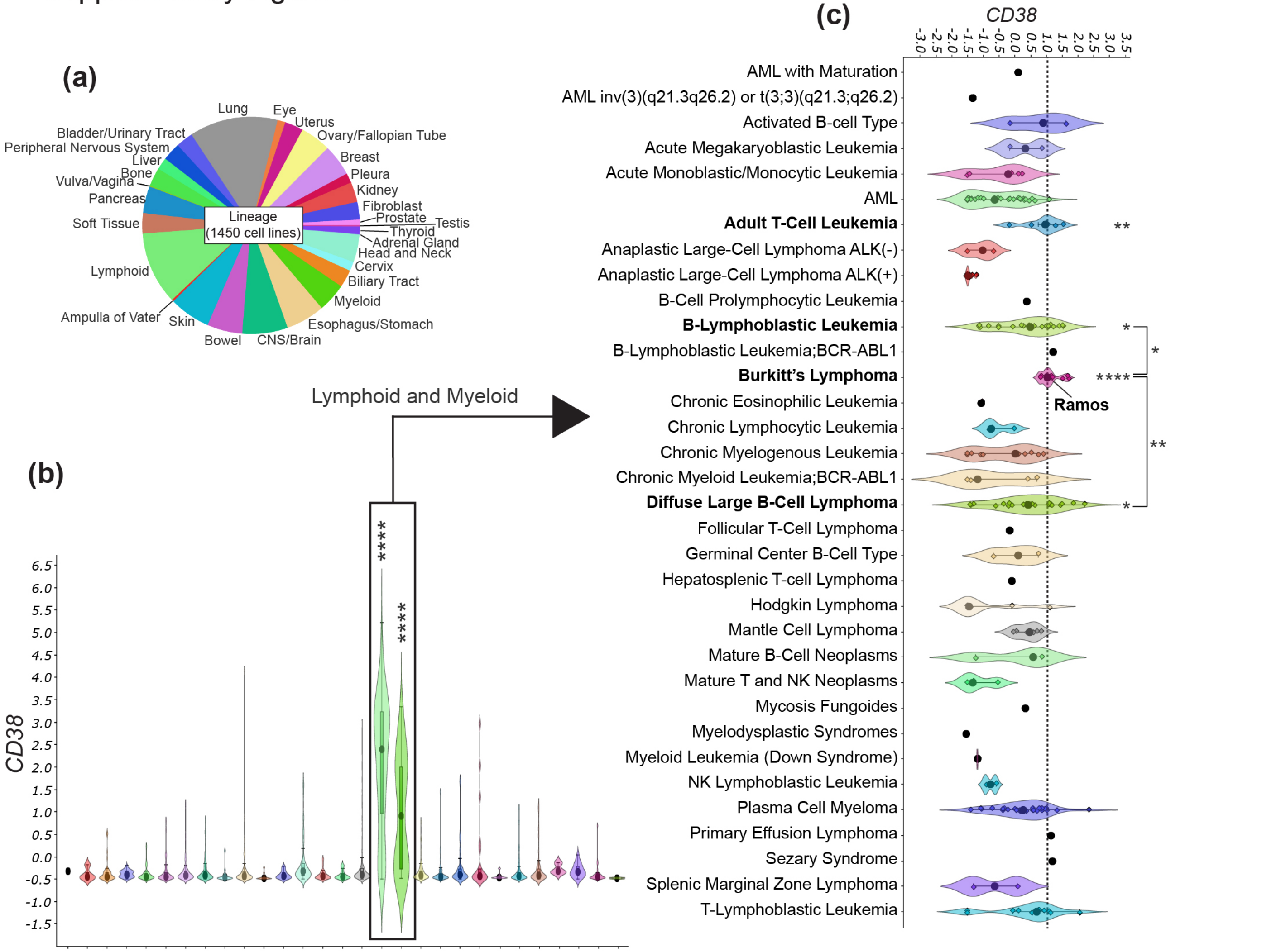

Supplementary Figure 3: Ramos cells as a model for CD38 mAb therapy studies. (a) Pie chart shows the lineage distribution of the 1450 cell lines contained in the dataset used for these analyses. **(b)** Violin plot shows CD38 gene expression in the cell line samples grouped by lineage. **(c)** Violin plot shows CD38 gene expression in the cell line samples contained in the lymphoid and myeloid lineages and analyzed according to disease subtypes. The dashed line indicates the median in the Burkitt Lymphoma disease subtype. In **(b)** and **(c)** the darker circles indicate the median value for each group of samples. In **(c)** each diamonds indicate samples. Asterisks without connecting lines above each group indicate the significance of that group when compared against all other groups in the dataset. Additionally, asterisks with lines connecting specific groups represent the results of further analyses conducted among those groups that showed significant differences in the initial test. The significance levels in **(b)** and **(c)** were calculated with Mann-Whitney and one-way ANOVA with post-hoc Tukey tests and denoted as *P < 0.05; **P < 0.01; ****P < 0.0001. Non-significance is not indicated.

Supplementary Figure 4

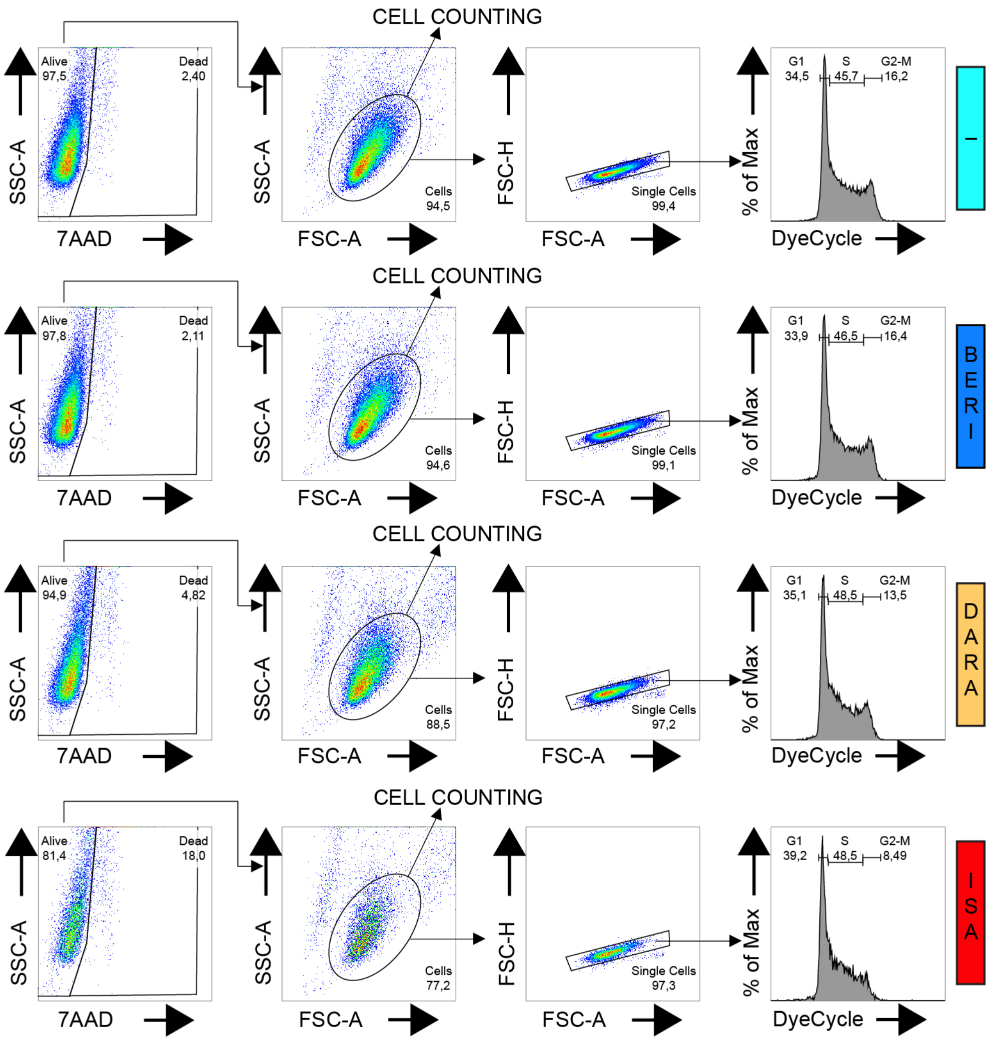

Supplementary Figure 4: Representative flow cytometry gating strategy for Ramos cells. Representative flow cytometry gating strategy of Ramos cells for assessing percentage of dead cells as 7AAD+, proliferation as number of cells (cell counting) and cell cycle as the distribution of the Vibrant DyeCycle in cells untreated (–) or treated with beriglobin, DARA or ISA.

Supplementary Figure 5

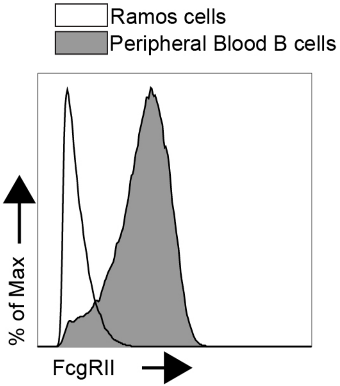

Supplementary Figure 5: FcγRII expression on Ramos and peripheral blood B cells. Flow cytometry histograms show FcγRII expression in Ramos (solid black line) and peripheral blood B (gray filled) cells.

Supplementary Figure 6

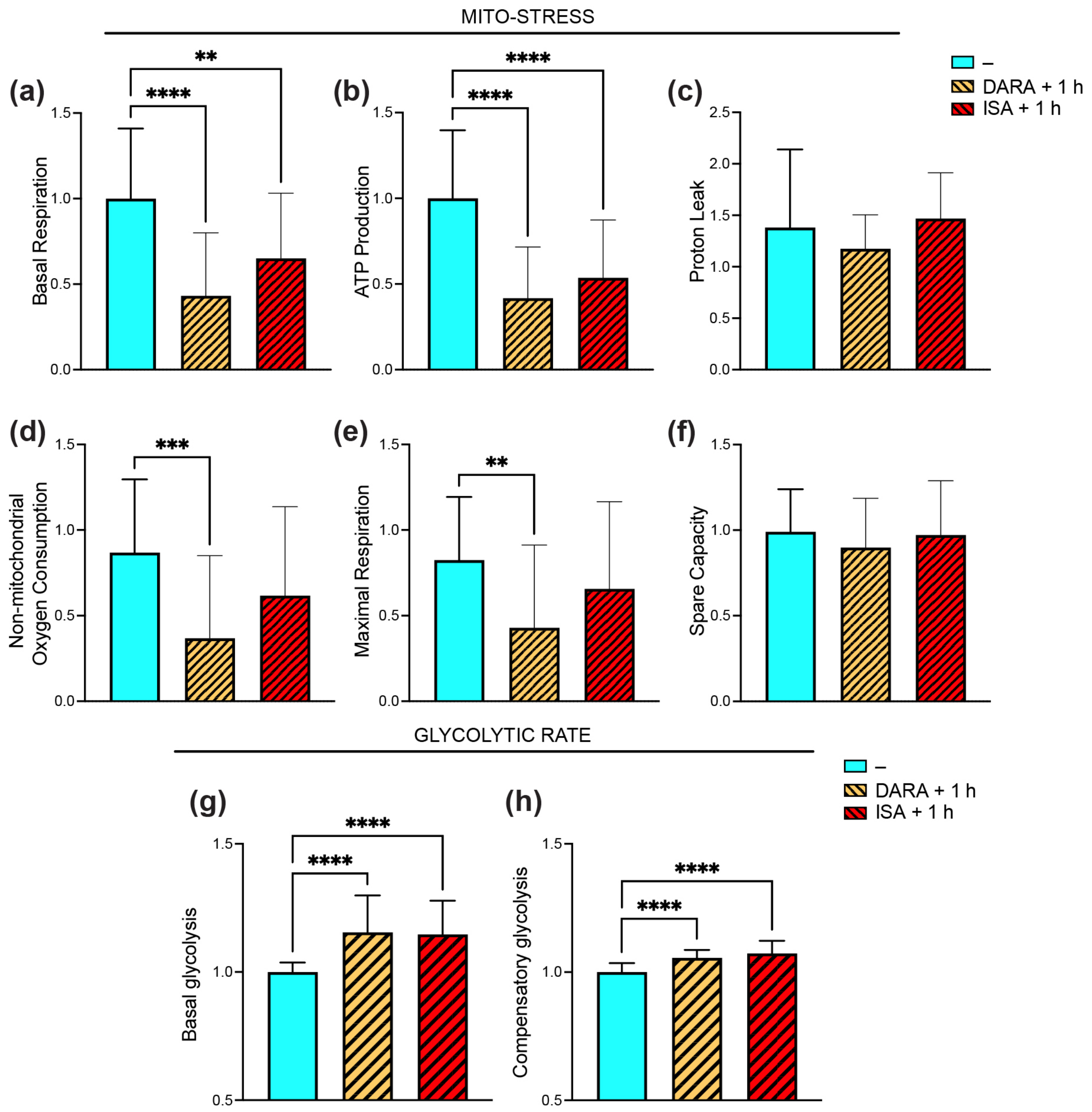

Supplementary Figure 6: Metabolic changes in pBL cells after two h treatment with DARA and ISA. Ramos cells, untreated (–) or treated with DARA or ISA for two h were assessed for metabolic changes involving mitochondrial respiration (Mito-stress) and glycolysis (Glycolytic rate). **(a)** presents the basal respiration rates, while **(b)** shows ATP production. **(c)** and **(d)** illustrate proton leak and non-mitochondrial oxygen consumption, respectively. The maximal respiration rate and spare respiratory capacity were depicted in **(e)** and **(f)**. Further, glycolytic function is assessed in **(g)** and **(h)** where basal and compensatory glycolysis levels were shown. Statistical significance in this figure was calculated with the one-way ANOVA with post-hoc Tukey test and denoted as ***P* < 0.01; ****P* < 0.001; *****P* < 0.0001. Data are presented as mean ± SD. Non-significance is not indicated in the figure. Data in **a-f** represent data from three independent experiments, with results normalized and combined. **g** and **h** represent data from two independent experiments, with results normalized and combined.

Supplementary Figure 7

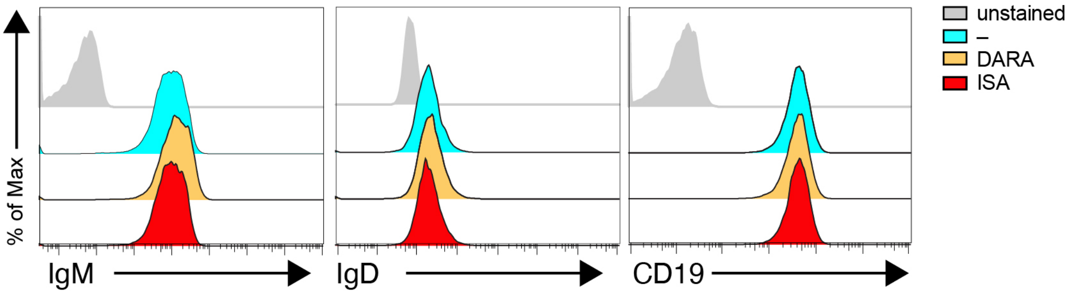

Supplementary Figure 7: IgM, IgD and CD19 surface expression upon treatment with DARA and ISA. IgM, IgD and CD19 surface expression levels on Ramos cells were assessed by flow cytometry upon 30 min treatment with DARA and ISA and compared with unstained and untreated (–) cells as controls.

Supplementary Figure 8

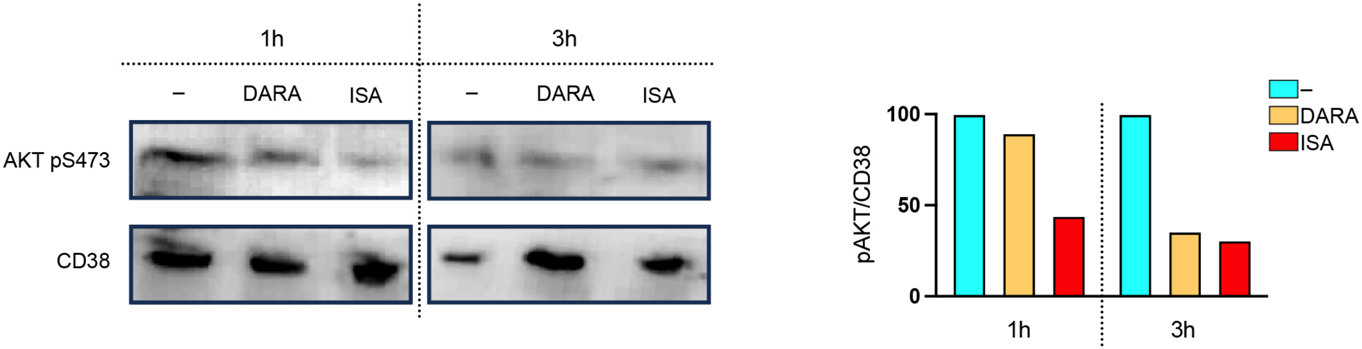

Supplementary Figure 8: Immunoblot analysis of pAKT in pBL cells upon treatment with DARA and ISA. The left panel shows the immunoblot results for AKT pS473 and CD38 at 1 hour and 3 hours of treatment with DARA, ISAand untreated control (–). The right panel quantifies the pAKT/CD38 ratio, normalized to untreated controls (blue bar). This figure represents data from two independent experiments.
